# Effects of maca (*Lepidium meyenii*) on nutrient digestibility and major nutrient transporters in rats fed a high‐fat diet

**DOI:** 10.1002/fsn3.2545

**Published:** 2021-08-31

**Authors:** Nurhan Sahin, Cemal Orhan, Hasan Gencoglu, Besir Er, Ibrahim H. Ozercan, James R. Komorowski, Kazim Sahin

**Affiliations:** ^1^ Department of Animal Nutrition and Nutritional Disorders Faculty of Veterinary Medicine Firat University Elazig Turkey; ^2^ Department of Biology Faculty of Science Firat University Elazig Turkey; ^3^ Department of Pathology Faculty of Medicine Firat University Elazig Turkey; ^4^ Research and Development Nutrition21 LLC Harrison NY USA

**Keywords:** digestibility, high‐fat diet, maca, nutrient transporters, rat

## Abstract

**Scope:**

This study was carried out to investigate the efficacy of a new combination of root extracts of the *Lepidium meyenii* (maca) plant, known for its nutritional and energizing features as well as its antioxidant properties, on nutrient digestibility and nutrient transporters expression.

**Methods and results:**

A total of 28 Sprague‐Dawley rats (8‐week‐old) were divided into four groups: (i) control, (ii) *Lepidium m*., (iii) high‐fat diet (HFD), and (iv) HFD+*Lepidium m*. Maca was given to the rats as a powdered combination of the plant roots with a daily dose of 40 mg per kg BW. Maca administration significantly increased the digestibility of dry matter (DM), organic matter (OM), crude protein (CP), and ether extract (EE), and some nutrient transporter (Pept1/2, Fatp1, Glut1/2, and Sglt1)‐expressions compared with non‐treated control and HFD groups in the jejunum and ileum tissues (*p* < .0001).

**Conclusions:**

Maca supplementation improved the digestibility of nutrients and expressions of nutrient transporters in the small intestine of the rats. These results indicate the positive communication between maca consumption and nutrient absorption in the small intestines of the animals.

## Graphical abstract

Maca supplementation to the basal and HFD diets of the rats improved digestibility of DM, OM, CP, and EE along with the enhancement of Pept1/2, Fatp1, Glut1/2, and Sglt1 expressions in the jejunum and ileum of the rats.

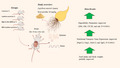

## 1 INTRODUCTION

Nutritional complementary choices that could make significant health interventions in the eating habits of humans and companion animals are getting more attention in the prevention of chronic diseases (Thompson et al., 2014; Willett et al., 2006). Even though people following a balanced diet do not generally need to take additional nutrients, many people also use supplements to meet their right micronutrient needs to be protected from chronic diseases with the versatile effects of the developing world (Tontisirin et al., 2002). Obesity is an important relationship between chronic medical conditions such as cardiovascular diseases, diabetes mellitus, hypertension, and various cancers, making it a health and financial burden from the past to the present (Hurt et al., 2010; Tremmel et al., 2017). Westernized diets contain high saturated fats and cholesterol, low fiber, as well as high sugar and salt, which is known to increase the risk of cardiovascular diseases (CVDs) (Statovci et al., 2017). The preventive roles of plant‐based phytochemicals in obesity and related CVDs, together with supportive metabolic, molecular approaches, have created awareness about this field in recent years, enabling novel insights (Gencoglu et al., 2017).

*Lepidium meyenii* (maca), a plant from the Brassicaceae family, is an edible medicinal plant in Peruvian tradition and has been used as a food source for centuries (Gonzales, 2012). In recent years, bioactive compounds such as fatty acids, glucosinolates, isothiocyanates, phenols, and polysaccharides in the maca plant have been measured using different methods and reported to be effective in various metabolisms (Korkmaz, 2018). Besides the macaenes, macamides are the specific maca ingredients, and the total macamide fraction (TMM) has been shown to have antioxidant and significant antitumor efficacy against the five different cancer cell lines (Fu et al., 2021). However, a novel identified polyunsaturated macamide derivative of *Lepidium meyenii* was reported to relieve dextran sulfate sodium‐induced colitis in mice as evidence of the beneficial efficacy of Lepidium meyenii in mice intestines (Zha et al., 2021). The maca's phenolic compounds and polysaccharides represent their antioxidant effects against oxidative stress via inhibiting the free radicals (Silva Leitão Peres et al., 2020). Yet, the claims about the centuries‐old health benefits of maca were partially supported by functional studies and clinical trials (Beharry & Heinrich, 2018; Silva Leitão Peres et al., 2020), and the role of nutrient‐transporter proteins in the intestines has not been elucidated yet for its activity. It has been exposed that a metabolic response to different fat sources containing a high‐fat diet (HFD) was linked with alterations in the hepatic peptidases, which are crucial in regulating glucose metabolism and oxidative stress (Domínguez‐Vías et al., 2020). In a recent maca study with HFD rats, it has been shown that maca is a potential SIRT1 activator in the liver and can significantly increase IRS1 levels in the visceral adipose tissue (Gencoglu, 2020). The apparent digestibility of bone minerals has been reported to significantly decrease in HFD diets, leading to reduced bone density (Frommelt et al., 2014). Prebiotics have health‐enhancing effects due to the modulation of the human colon microbiota, which is selectively combined with living microbial species called probiotics in the human intestine; therefore, studies on polysaccharides have focused on probiotic activities in the intestines (Markowiak & Śliżewska, 2017). In a recent study, a neutral polysaccharide obtained from the maca roots induced a higher growth of probiotics and short‐chain fatty acids than inulin and showed prebiotic properties, while it also improved the anti‐inflammatory effects (Lee et al., 2020).

In the present study, we aimed to determine the effectiveness of maca on nutrient digestibility and the mRNA changes in nutrient‐transporter proteins, including glucose transporters (Glut1/Glut2), peptide transporters (Pept1/Pept2), fatty acid transporter 1 (Fatp1), along with the sodium/glucose cotransporter 1 (Sglt1) in the intestinal tissues of HFD fed rats, for achieving novel molecular interaction mechanisms and also preventive choices.

## 2 MATERIALS AND METHODS

### 2.1 Animals

A total of 28 young adult male Sprague‐Dawley rats (*n* = 7 in each group, 12 weeks old, and weighing 200 ± 20 g) were housed in individual cages under controlled temperature (22°C) and a 12 h’ light–dark cycle and provided with ad‐libitum standard and high‐fat diets and tap water (Table 1). The rats were purchased from the Firat University Animal Experimental Unit, Elazig, Turkey. The animal protocol performed in this study was assessed and approved by the Firat University Animal Experiments Ethics Committee (Number: 2019/09/88) and conducted according to the National Institutes of Health Guidelines for the Care and Use of Laboratory Animals.

**TABLE 1:** Composition of diets (g/kg) and chemical analysis

	Control	HFD
Casein	200.0	200.0
Corn starch	579.5	150.0
Sucrose	50.0	149.5
Soy oil	70.0	–
Beef tallow	–	400.0
Cellulose	50.0	50.0
Vitamin‐mineral mixture^a^	45.0	45.0
L‐cysteine	3.0	3.0
Choline bi‐tartrate	2.5	2.5
Chemical analysis		
Metabolic energy (kcal/kg)	3802	4811 kcal/kg
Crude protein (%)	17.8	17.8
Ether extract (%)	7.0	39.8
Crude cellulose (%)	4.9	4.9
Ash (%)	4.3	4.3

Note

Mineral Premix (g/kg mixture): anhydrous calcium carbonate 357, monobasic potassium phosphate 196, sodium chloride 74, potassium sulfate 46.6, potassium citrate monohydrate 70.78, ferric citrate 6.06, zinc carbonate 1.65, manganous carbonate 0.63, cupric carbonate 0.30, potassium iodate 0.01, anhydrous sodium selenate 0.01025.

^a^ Vitamin Premix (g/kg mixture): Niacin 3, Ca‐pantothenate 1.6, pyridoxine‐HCl 0.7, thiamine HCl 0.6, riboflavin 0.6, folic acid 0.2, D‐Biotin 0.02, vitamin B12 (0.1% cyanocobalamin in mannitol) 2.5, vitamin E (all‐rac‐α‐tocopheryl acetate, 500 IU/g) 15, vitamin A (all‐trans‐retinyl palmitate, 500,000 IU/g) 0.80, Vitamin D3 (cholecalciferol, 400,000 IU/g) 0.25, Vitamin K (phylloquinone) 0.075.

### 2.2 Study design

The rats were randomly divided into four groups as follows: (i) control group, rats fed with a standard diet. (ii) *Lepidium meyenii* group, in addition to the fed standard diet, rats were received maca powder extract at a daily dose of 40 mg/kg BW via intragastric gavage for 8 weeks. (iii) high‐fat diet group (HFD), rats were fed with a high‐fat diet. (iv) HFD + *Lepidium meyenii* group, in addition to a fed high‐fat diet, rats were given a daily dose of 40 mg/kg / BW of maca powder extract by intragastric gavage for 8 weeks. The dosage of maca was selected according to previous studies (Gencoglu, 2020; Vásquez‐Velásquez et al., 2020). The nutritional supplement used in this study was an effective amount of maca combination (*Lepidium meyenii* Linn.). The maca roots powder compositions consisted of a synergistic compound of black and red maca in a ratio of about 4:1 to about 1:4. (Nutrition 21). The control and HFD groups were also treated with 1 ml of drinking water as a vehicle for each rat via intragastric gavage to equalize the gavage stress among the groups. The gavage procedures were carried out simultaneously the day without anesthesia during the 8 weeks. The composition of basal and HFD diets was given in Table 1.

### 2.3 Sample collection

Fecal samples were collected twice daily for 5 days from all rats after maca administration. The feces collection was started at 09.00 h on the morning of day 56 of the collection period and ended at 09.00 h on day 60 of the study. At the end of the 8‐week study, all the rats were sacrificed with decapitation. Then, the abdominal cavity of rats was opened, and the small intestine was removed to isolate 2–3 cm segments of the duodenum, jejunum, and ileum. Tissues were rapidly put on the dry ice and then frozen at −80°C as well for the determination of mRNAs expression.

### 2.4 Sample preparation

Individual fecal samples were mixed, homogenized, and pooled (on a weight basis) per rat. All fecal samples were composited and oven‐dried at 60°C for 48 h, were ground, and sub‐sampled (1 g) for chemical analyses. The ground samples of feed and feces were analyzed in triplicate for dry matter (DM), crude protein (CP), ether extract (EE), and ash according to the procedures of the AOAC (1990) (AOAC, 1990). Chromic oxide (Cr2O3, 0.2% of diet) was added as an indigestible marker (Sahin et al., 2006).

### 2.5 Determination of chromium concentration

The concentration of chromium in feed and feces was determined using an atomic absorption spectrophotometer (AAS, Perkin‐Elmer; Analyst 800) containing a graphite furnace and tubes. For this purpose, 0.3 g of feed and feces weighed into Teflon digestion vessel were digested in 5 ml of 65% (v/v) spectra pure HNO3 (Merck) in the Speedvawe MWS‐2 Microwave Digestion System (Berghoff). All measurements were evaluated at a wavelength of 357.9 nm. Calibration was performed by preparing five standards (0.5, 1.0, 2, 4, 8, and 10.0 μg/L) using a chromium stock standard of 1.000 μg/mL concentration (Merck). The mean recovery of the reference material for the samples was 96%. The detection limit (LOD) for the Cr analysis method was calculated by the equation LOD = 3*s*/*m* (*s*‐standard deviation of the measurements of the blank; the *m*‐the slope of the calibration curve). The precision of the method was verified as a 4.6% CV.

### 2.6 Calculation of nutrient digestibility

Nutrient digestibility was formulated as follow:

100‐[100×(%Cr in diet/%Cr in feces)×(%nutrient in feces/%nutrient in diet)].

### 2.7 Real‐time quantitative PCR

RNeasy Mini kits (Qiagen) were used for the intestinal tissue homogenizations and total RNA extractions, considering the manufacturer's extraction guidelines. RNA yield was determined via NanoDrop (MaestroGen). RNA samples were either stored at −80°C for long‐term storage or immediately kept on ice for cDNA synthesis. For cDNA synthesis, 2 µg of total RNA was reverse transcribed by TaqMan1 Reverse Transcription reagents (Qiagen). Real‐time quantitative RT‐qPCR was done on cDNA aliquots with a SYBR Green PCR Master Mix (Catalog no. 330620, Qiagen) to quantitatively assess the gene expressions on Rotor‐Gene Q (Qiagen). Glyceraldehyde‐3‐phosphate dehydrogenase (Gapdh) was used as the internal control. Reactions were performed in triplicates with 2 µl primer pair, 5 µl SYBR green master mix, 1 µl RNA‐free water, and 2 µl cDNA templates. The primers used for the amplification are listed in Table 2. PCR was completed with the following conditions: initial denaturation at 95°C for 15 s, 40 cycles, annealing at 60°C for 15 s, and extension together at 70°C for 30 s. Each PCR was made in triplicate, and the mean *C*t value was used for statistical analysis. mRNA expressions were standardized using the Gapdh expression levels and then normalized according to the control group.

**TABLE 2:** RT‐qPCR primer sequences

Gene	Gene Bank #	Sense	Antisense
*Slc15a1*	NM_057121.2	CTTCGACAAACAGTGGGCTGA	GCAAGGACTCTGTGGTGGAGG
*Slc15a2*	NM_031672.2	TGCAGTTGCAGCACTTGTCG	GCTGACGGACTCCACCAAGA
*Slc27a1*	NM_053580.2	TGCGAGAACCCGTGAGGAA	CGATACGCAGAAAGCGCCAG
*Slc2a1*	NM_138827.2	TCTCTGTCGGGGGCATGATT	AACCCATAAGCACGGCAGAC
*Slc2a2*	NM_012879.2	AGTCACACCAGCACATACGA	TGGCTTTGATCCTTCCGAGT
*Slc5a1*	NM_013033.2	AAGCGATTTGGAGGCAAGCG	CCAGTCCCCCTGTGATGGTG
*Gapdh*	NM_017008.4	GGTTACCAGGGCTGCCTTCT	CTTCCCATTCTCAGCCTTGACT

### 2.8 Statistical procedures

The sample size was calculated with a power of 85%, and the *p* < .05 was reflected to be statistically significant. One‐way ANOVA and Tukey's multiple comparison tests were used for evaluating the differences between means as appropriate and the data plotted with software (GraphPad Software, Inc.).

## 3 RESULTS

### 3.1 Feed intake and body weight changes

**FIGURE 1:**
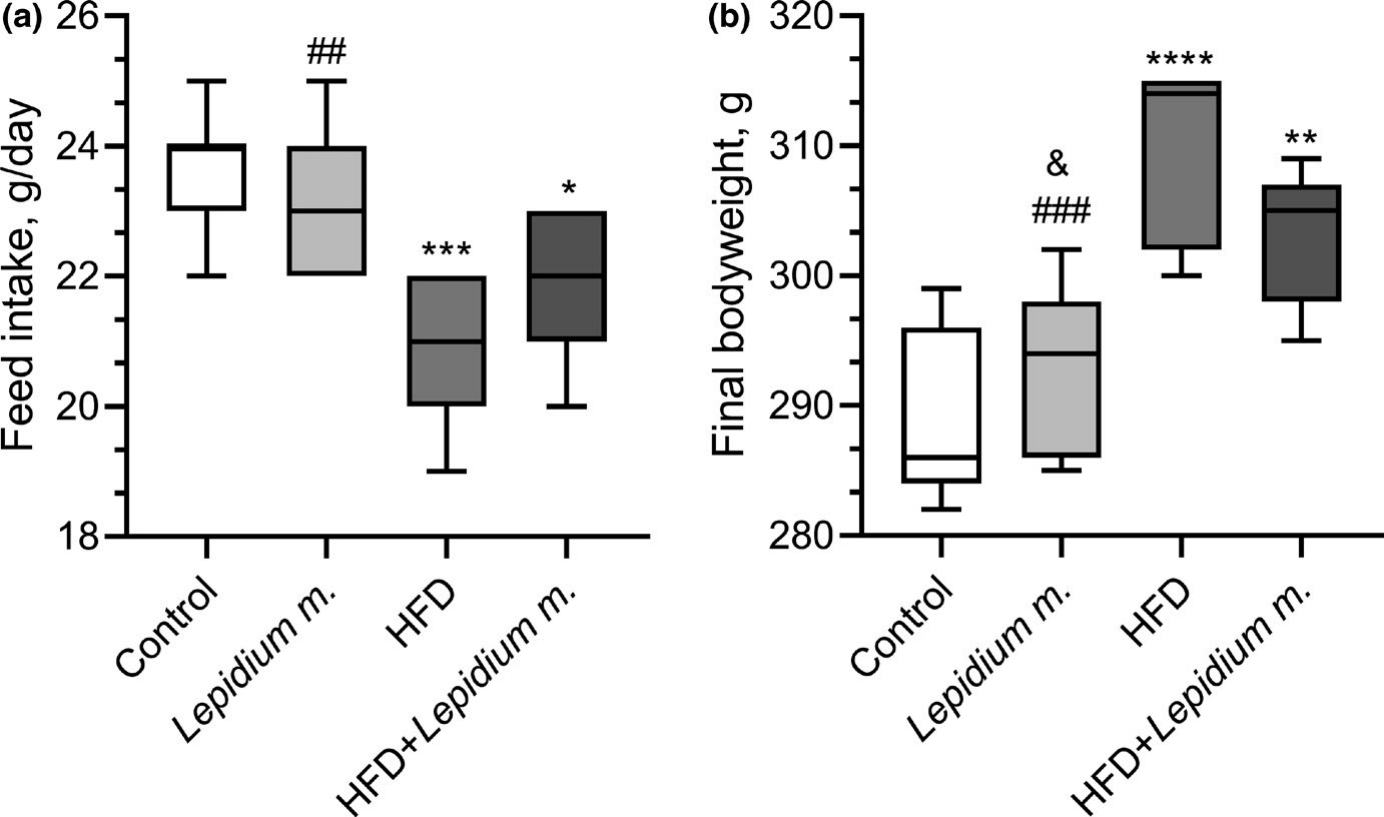
Effects of *Lepidium meyenii* (Maca) on feed intake (a) and body weight change (b). Maca Root Extract (40 mg/kg/day, oral gavage), HFD; high‐fat diet. Statistical significance between groups is shown by *p < .05, **p < .01, ***p < .001, and ****p < .0001 compared with control group; ^#^ *p* < .05, ^##^ *p* < .01, and ^###^ *p* < .001 compared with HFD group; ^&^ *p* < .05 compared with HFD + *Lepidium meyenii* group

Feed consumption in groups is presented in Figure 1a. Accordingly, the average feed consumption did not change in the *Lepidium meyenii* group compared with the control group (*p* > .05), while lower feed consumption was seen in HFD and HFD + *Lepidium meyenii* groups compared with the control. The significances were as follows: control versus HFD; *p* = .0002, control versus HFD + *Lepidium m*.; *p* = .017, and *Lepidium m*. versus HFD; *p* = .002. Also, there was a 12.1% decrease between control and HFD, a 7.9% decrease between control and HFD + *Lepidium m*., and a 4.8% increase between HFD and HFD + *Lepidium m*.

While the final body weights in the *Lepidium meyenii* group did not change compared with the control group, a significant increase in the body weight was found in the HFD and HFD + *Lepidium meyenii* groups compared with the control group and *Lepidium meyenii* group (Figure 1b). The significances were as follows: control versus HFD; *p* < .0001, control versus HFD + *Lepidium m*.; *p* = .002, *Lepidium m*. versus HFD; *p* = .0002, and *Lepidium m*. versus HFD + *Lepidium m*.; *p* = .037. There was a 7.2% increase in the HFD and a 4.7% increase in the HFD + *Lepidium m*., compared with the control and a 2.8% decrease in the HFD compared with HFD + *Lepidium m*.

### 3.2 Nutrient digestibility

**FIGURE 2:**
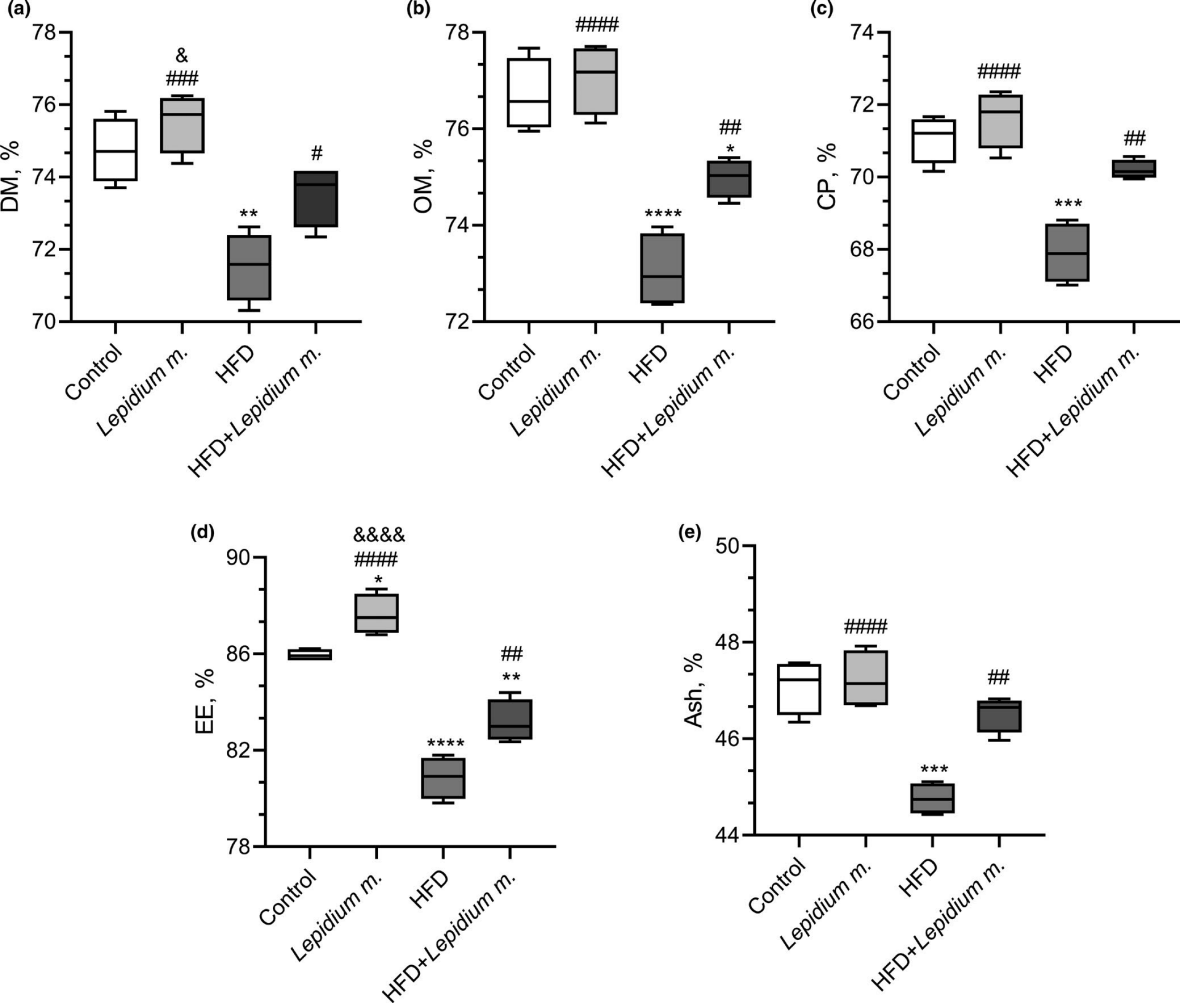
Effects of *Lepidium meyenii* (Maca) on dry matter (DM, panel a), organic matter (OM, panel b), crude protein (CP, panel c), ether extract (EE, Panel d) and ash (Panel e). HFD; high‐fat diet. Statistical significance between groups is shown as: *p < .05, **p < .01, ***p < .001, and ****p < .0001 compared with control group; ^#^ *p* < .05, ^##^ *p* < .01, ^###^ *p* < .001, and ^####^ *p* < .0001, compared with HFD group, and ^&^ *p* < .05; ^&&^ *p* < .01; ^&&&^ *p* < .001; and ^&&&&^ *p* < .0001 compared with HFD + *Lepidium meyenii* group

The digestibility of DM, OM, CP, EE, and ash in the rats is shown in Figure 2a–e. As shown in Figure 2a–e, no significant changes in the digestibility of DM, OM, and CP were observed in rats fed with a standard diet (*p* > .05). HFD intake impaired the nutrient digestibility in rats by significantly decreasing the DM (*p* = .001), OM (*p* < .0001), CP (*p* = .0001), EE (*p* < .0001), and ash (*p* = .0001). The adverse effects of HFD on the nutrient digestibility variables were evident, as reflected by decreased DM (4.3%), OM (4.7%), CP (4.5%), EE (5.9%), and ash (5.0%) compared with the control group. However, *Lepidium m*. supplementation improved digestibility of DM (*p* = .034), OM (*p* = .008), CP (*p* = .002), EE (*p* = .005), and ash (*p* = .001) of rats fed with an HFD. There were 2.8%, 2.6%, 3.4%, 2.9%, and 4.0% increases in the HFD + *Lepidium m*. compared with the HFD for the digestibility of DM, OM, CP, EE, and ash, respectively (Figure 2a–e).

### 3.3 Gene expressions of the nutrient transporters

**FIGURE 3:**
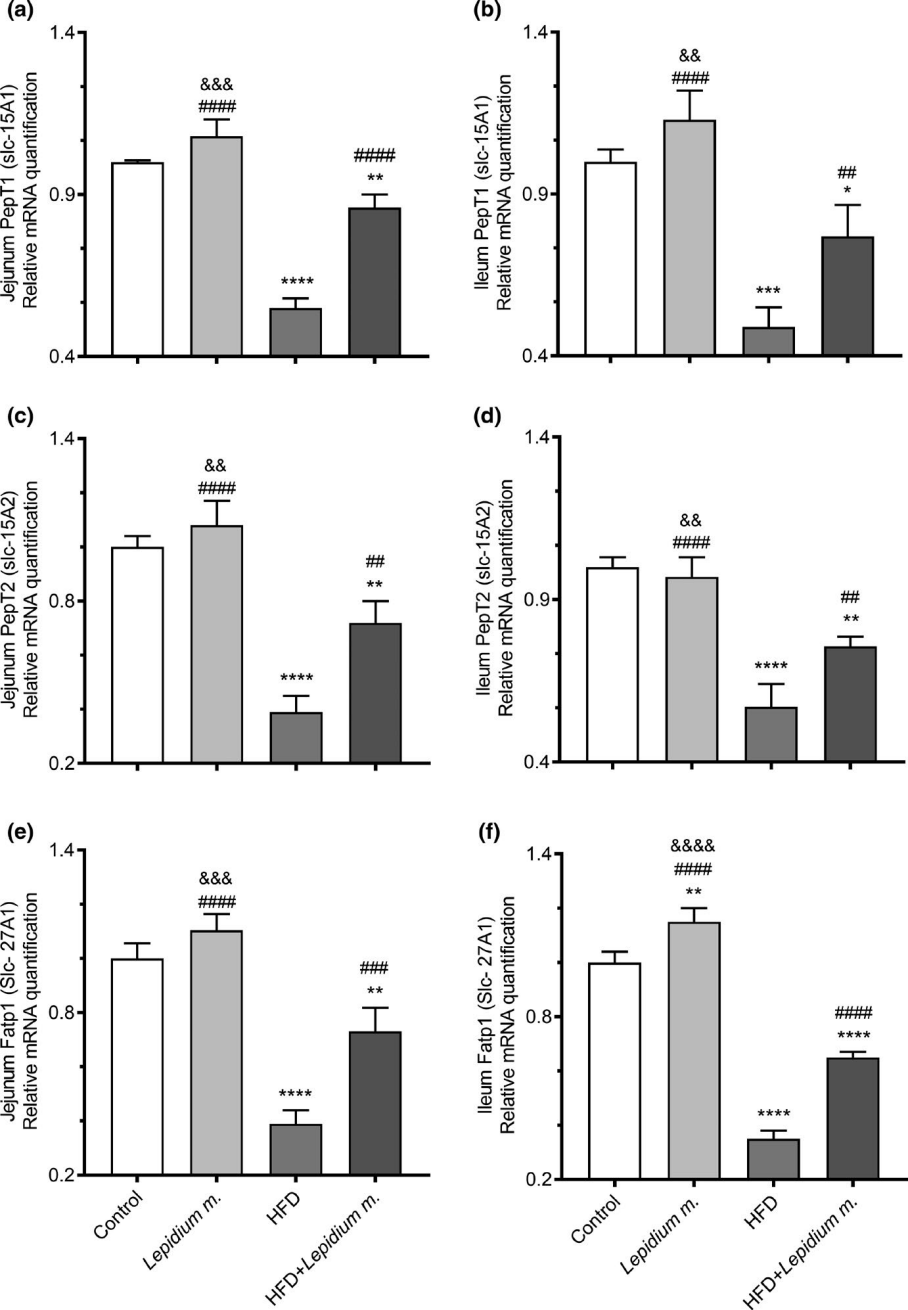
Effects of *Lepidium meyenii* (Maca) on rat jejunum Pept1 (a), ileum Pept1 (b), jejunum Pept2 (c), ileum Pept2 (d), jejunum Fatp1 (e), and ileum Fatpt1 (f), mRNA expressions (fold of control). Statistical significance between groups is shown as: *p < .05, **p < .01, ***p < .001, and ****p < .0001 compared with control group; ^##^ *p* < .01, ^###^ *p* < .001, and ^####^ *p* < .0001, compared with HFD group, and ^&&^ *p* < .01; ^&&&^ *p* < .001; and ^&&&&^ *p* < .0001 compared with HFD + *Lepidium meyenii* group

**FIGURE 4:**
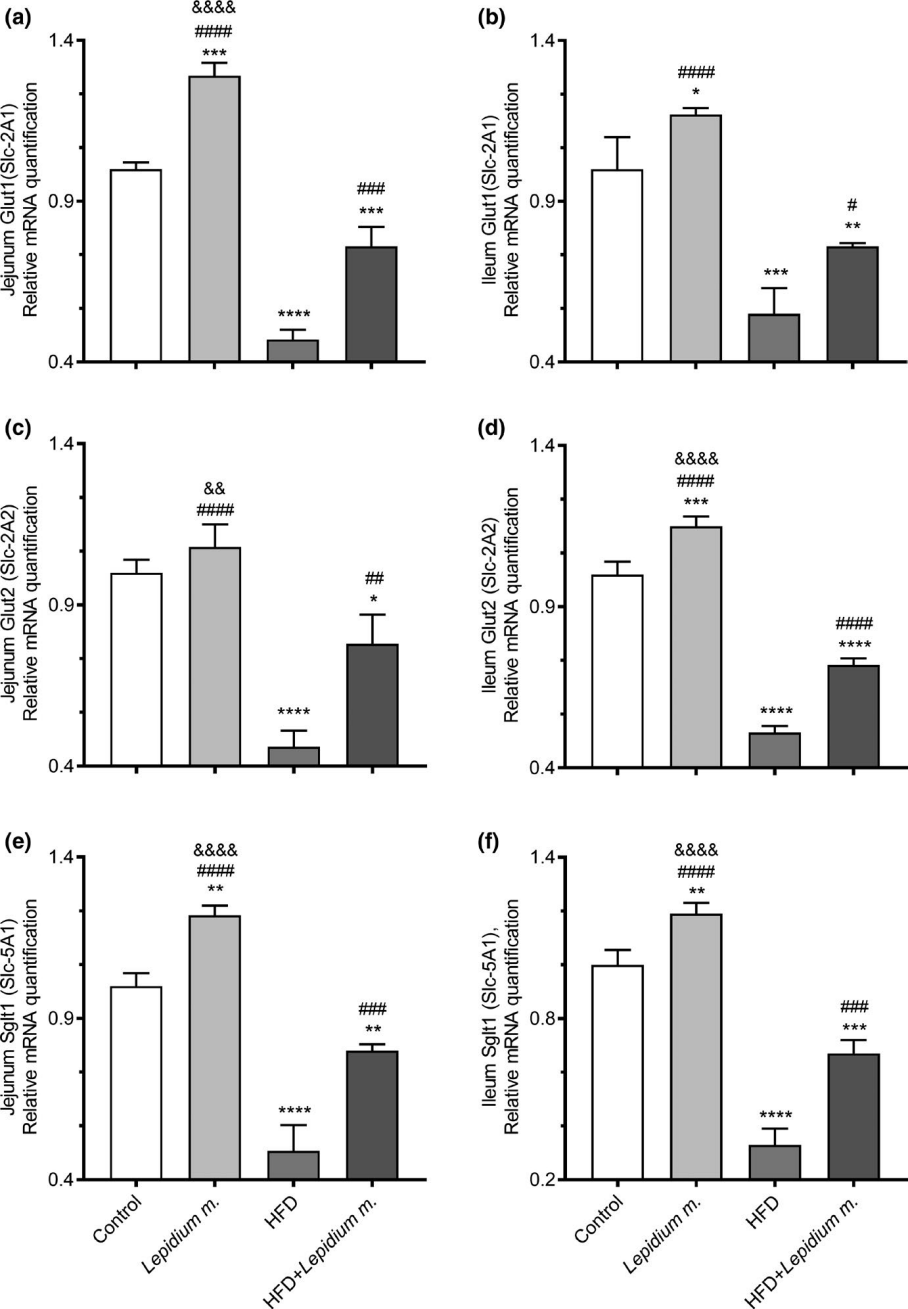
Effects of *Lepidium meyenii* (Maca) on rat jejunum Glut1 (a), ileum Glut1 (b), jejunum Glut2 (c), ileum Glut2 (d), jejunum SGLT1 (e), and ileum Sglt1 (f), mRNA expressions (fold of control). Statistical significance between groups is shown as: *p < .05, **p < .01, ***p < .001, and ****p < .0001 compared with control group; ^#^ *p* < .05, ^##^ *p* < .01, ^###^ *p* < .001, and ^####^ *p* < .0001, compared with HFD group and, ^&&^ *p* < .01; ^&&&&^ *p* < .0001 compared with HFD + *Lepidium meyenii* group

The jejunum and ileum Pept1, Pept2, and Fatp1, gene expression variations between the groups are presented in Figure 3a–f, and Glut1, Glut2, and Sglt1 in Figure 4a–f. There were 45% and 51% decreases in the HFD versus control (*p* < .0001 and *p* < .001), 14% and 23% decreases in the HFD + *Lepidium m*. compared with control (*p* < .01 and *p* < .05), and 56% and 57% increases in the HFD + *Lepidium m*. against the HFD for the jejunum (Figure 3a) and ileum (Figure 3b) Pept1 expressions (*p* < .0001 and *p* < .01). There were 61% and 43% decreases in the HFD compared with the control for the expressions of the jejunum and ileum Pept2 (Figure 3c,d). In Pept2 jejunum and ileum expressions, there were 61% and 43% decreases in HFD compared with the control (*p* < .0001), while 84% and 33% increases were found in the HFD + *Lepidium m*. compared with the HFD group (*p* < .01; Figure 3c,d). Moreover, in jejunum Fatp1, there was a 61% decrease in the HFD in comparison with control (*p* < .0001), a 27% decrease in the HFD + *Lepidium m*. when compared to control (*p* < .01), and an 88% increase in the HFD + *Lepidium m*. against the HFD (Figure 3e, *p* < .001). Additionally, in ileum Fatp1, there was a 65% decrease in the HFD in comparison to control (*p* < .0001), a 35% decrease in the HFD + *Lepidium m*. versus control (*p* < .0001), and an 86% increase in the HFD + *Lepidium m*. against the HFD (Figure 3f, *p* < .0001).

HFD intake affected glucose transporters by lowering the expressions of Glut1, Glut2, and Sglt1 in the jejunum and ileum of rats (Figure 4; *p* < .0001). The detrimental effects of HFD on jejunum and ileum Glut1 (*p* < .001 and *p* < .05), Glut2 (*p* < .01 and *p* < .0001), and Sglt1 (*p* < .001) were alleviated by *Lepidium m*. supplementation. Increases in the jejunum and ileum Glut1, Glut2, and Sglt1 expressions were found in the HFD + *Lepidium m*. group compared with the HFD group at 62% and 38%, 70% and 41%, and 63% and 103%, respectively (Figure 4a–f).

## 4 DISCUSSION

*Lepidium meyenii* (maca), native to the Andean region, is a valuable source of fiber and nutrients, including niacin, thiamine, riboflavin, zinc, manganese, copper, and iron, and contains bioactive compounds like macamides, that can benefit a healthy diet (Silva Leitão Peres et al., 2020; Zhu et al., 2020). Dried and minced maca roots have main dietary components, including 46%–74% of carbs and more than 10% of plant‐derived proteins, while it also comprises a healthful ratio of unsaturated to saturated fatty acid (53% vs. 40%, respectively), as well as high levels of linoleic and oleic acids (Wang & Zhu, 2019). Dietary components can dramatically change intestinal physiology and mainly regulate the intestines' barrier integrity (Chelakkot et al., 2018). The small intestine is a central digestive system component, which permits the body to break down and absorb the vital nutrients that allow it to work at the highest capacity (Collins et al., 2021).

The regulation of nutrient transporters in the intestinal lumen of the gastrointestinal tract (GI) is not fully understood yet. In the present study, maca supplementation did not statistically improve feed intake and BW gain, whereas significantly enhanced the digestibility of DM, OM, CP, EE, and ash levels along with a distinct up‐regulation of peptide transporters (Pept1/2), fatty acid transporter (Fatp1), glucose transporters (Glut1/2), and sodium‐dependent glucose transporter (Sglt1) expressions in the jejunum and ileum of the rats. Jejunum refers to the part of the small intestine that leaves the duodenum to one side, leading to the ileum, which is the primary purpose of the jejunum is to absorb monosaccharides, amino acids, and fatty acids, while the ileum absorbs the remnant nutrients that were not absorbed by the duodenum or jejunum, especially vitamin B12 and bile acids to be recycled (Collins et al., 2021). The glucose transporter families (Glut) and sodium‐dependent glucose transporters (Sglt) are responsible for the absorption of glucose, while the peptide transporters 1 and 2 are the members of the proton‐coupled oligopeptide transporter family and the Fatp1 mediates skeletal muscle cell fatty acid import (Byers et al., 2017; Guitart et al., 2014; Zwarycz & Wong, 2013). In a recent study, an HFD fed mouse with thyroid disorders for 16 weeks reported a significant reduction in Glut2, Pept1, and fatty acid translocase (FAT/CD36) expressions, indicating that HFD may impair nutrient intake in the small intestines (Torelli Hijo et al., 2019). *Lepidium meyenii* was suggested to be used as a natural antioxidant agent, which would be helpful in maintaining a balance between oxidants and antioxidants (Vecera et al., 2007) and could be a functional food consistently to our results. Maca supplementation was shown to decrease insulin levels while increasing IRS1, leptin, and antioxidant effective SIRT1 levels in rats fed a high‐fat diet (Gencoglu, 2020). Pept1 and Pept2 (SLC15A1 and SLC15A2) are H+‐coupled oligopeptide symporters, and current studies on the modulation of these genes in inflammatory gut diseases were suggested to provide useful data on the bioavailability choices as oral Pept medicine substrates (Ingersoll et al., 2011; Smith et al., 2013). In the present study, mRNA expressions of Pept1/2, Fatp1, Glut1/2, and Sglt1 levels increased in all maca‐given groups compared to those who did not receive the supplement in agreement with the study as mentioned earlier. In a similar manner to our study, in the HFD diet given mice intestines, the Glut2, Pept1, and membrane receptor FAT‐CD6 levels decreased, whereas Glut5 and Fatp4 remain unchanged (Losacco et al., 2018).

## 5 CONCLUSIONS

The present study showed the decent improving capacity of a novel form of *Lepidium meyenii* (maca) root powder composition on the feed intake, nutrient digestibility of DM, CP, OM, EE, and ash parameters, along with the gene expressions of the primary nutrient transporters Pept1, Pept2, Fatp1, Glut1, Glut2, and Sglt1, in the jejunum and ileum of the rats, that were administered for 60 days. The results of this study could be considered in the investigation and identifying approaches for the prevention and/or managing basal or HFD‐related nutritional intestinal disorders.

## CONFLICT OF INTEREST

The authors declare no conflict of interest.

## AUTHOR CONTRIBUTION

**Nurhan Sahin:** Conceptualization (lead); Data curation (lead); Formal analysis (lead); Funding acquisition (lead); Methodology (equal); Project administration (lead); Writing‐original draft (equal); Writing‐review & editing (equal). **Cemal Orhan:** Data curation (equal); Formal analysis (equal); Methodology (equal). **Hasan Gencoglu:** Writing‐original draft (equal). **Besir Er:** Data curation (equal); Formal analysis (equal); Methodology (equal). **Ibrahim H Ozercan:** Formal analysis (equal). **James R Komorowski:** Writing‐review & editing (equal). **Kazim Sahin:** Methodology (equal); Writing‐review & editing (equal).

## DISCLOSURE STATEMENT

The authors of this study have no commercial or proprietary interest in any concept or product described in this article.

## Data Availability

The datasets analyzed in the current study are available from the corresponding author on reasonable request.

## References

[fsn32545-bib-0001] AOAC (1990). Official methods of analysis, 15th ed. Association of Official Analytical Chemists.

[fsn32545-bib-0002] Beharry, S. , & Heinrich, M. (2018). Is the hype around the reproductive health claims of maca (*Lepidium meyenii* Walp.) justified? Journal of Ethnopharmacology, 211, 126–170. 10.1016/j.jep.2017.08.003 28811221

[fsn32545-bib-0003] Byers, M. S. , Howard, C. , & Wang, X. (2017). Avian and mammalian facilitative glucose transporters. Microarrays, 6, 7. 10.3390/microarrays6020007 PMC5487954 28379195

[fsn32545-bib-0004] Chelakkot, C. , Ghim, J. , & Ryu, S. H. (2018). Mechanisms regulating intestinal barrier integrity and its pathological implications. Experimental & Molecular Medicine, 50, 1–9. 10.1038/s12276-018-0126-x PMC6095905 30115904

[fsn32545-bib-0005] Collins, J. T. , Nguyen, A. , & Badireddy, M. (2021). StatPearls. StatPearls Publishing. 29083773

[fsn32545-bib-0006] da Silva Leitão Peres, N. , Cabrera Parra Bortoluzzi, L. , Medeiros Marques, L. L. , Formigoni, M. , Fuchs, R. H. B. , Droval, A. A. , & Reitz Cardoso, F. A. (2020). Medicinal effects of Peruvian maca (*Lepidium meyenii*): A review. Food & Function, 11, 83–92. 31951246 10.1039/c9fo02732g

[fsn32545-bib-0007] Domínguez‐Vías, G. , Segarra, A. B. , Ramírez‐Sánchez, M. , & Prieto, I. (2020). The role of high fat diets and liver peptidase activity in the development of obesity and insulin resistance in wistar rats. Nutrients, 12, 636. 10.3390/nu12030636 PMC7146256 32121057

[fsn32545-bib-0008] Frommelt, L. , Bielohuby, M. , Stoehr, B. J. M. , Menhofer, D. , Bidlingmaier, M. , & Kienzle, E. (2014). Effects of low‐carbohydrate, high‐fat diets on apparent digestibility of minerals and trace elements in rats. Nutrition, 30, 869–875. 10.1016/j.nut.2013.11.017 24985005

[fsn32545-bib-0009] Fu, L. , Wei, J. , Gao, Y. , & Chen, R. (2021). Antioxidant and antitumoral activities of isolated macamide and macaene fractions from *Lepidium meyenii* (Maca). Talanta, 221, 121635. 10.1016/j.talanta.2020.121635 33076155

[fsn32545-bib-0010] Gencoglu, H. (2020). Maca modulates fat and liver energy metabolism markers insulin, IRS1, leptin, and SIRT1 in rats fed normal and high‐fat diets. Archives of Physiology and Biochemistry, 19, 1–7. 10.1080/13813455.2020.1821064 32951476

[fsn32545-bib-0011] Gencoglu, H. , Orhan, C. , & Sahin, K. (2017). Phytochemical therapies in vascular functioning: A molecular approach. Current Vascular Pharmacology, 15, 327–338. 10.2174/1570161115666170105122616 28056757

[fsn32545-bib-0012] Gonzales, G. F. (2012). Ethnobiology and ethnopharmacology of *Lepidium meyenii* (Maca), a plant from the Peruvian Highlands. Evidence‐Based Complementary and Alternative Medicine: Ecam, 2012, 193496. 21977053 10.1155/2012/193496 PMC3184420

[fsn32545-bib-0013] Guitart, M. , Osorio‐Conles, Ó. , Pentinat, T. , Cebrià, J. , García‐Villoria, J. , Sala, D. , Sebastián, D. , Zorzano, A. , Ribes, A. , Jiménez‐Chillarón, J. C. , García‐Martínez, C. , & Gómez‐Foix, A. M. (2014). Fatty acid transport protein 1 (FATP1) localizes in mitochondria in mouse skeletal muscle and regulates lipid and ketone body disposal. PLoS One, 9, e98109. 10.1371/journal.pone.0098109 24858472 PMC4032244

[fsn32545-bib-0014] Hurt, R. T. , Kulisek, C. , Buchanan, L. A. , & McClave, S. A. (2010). The obesity epidemic: Challenges, health initiatives, and implications for gastroenterologists. Gastroenterology & Hepatology, 6, 780–792. 21301632 PMC3033553

[fsn32545-bib-0015] Ingersoll, S. A. , Ayyadurai, S. , Charania, M. A. , Laroui, H. , Yan, Y. , & Merlin, D. (2011). The role and pathophysiological relevance of membrane transporter PepT1 in intestinal inflammation and inflammatory bowel disease. American Journal of Physiology‐Gastrointestinal and Liver Physiology, 302, G484–G492. 10.1152/ajpgi.00477.2011 22194420 PMC3311434

[fsn32545-bib-0016] Korkmaz, S. (2018). Antioxidants in Foods and Its Applications. IntechOpen.

[fsn32545-bib-0017] Lee, Y.‐K. , Jung, S. K. , & Chang, Y. H. (2020). Rheological properties of a neutral polysaccharide extracted from maca (*Lepidium meyenii* Walp.) roots with prebiotic and anti‐inflammatory activities. International Journal of Biological Macromolecules, 152, 757–765. 10.1016/j.ijbiomac.2020.02.307 32114169

[fsn32545-bib-0018] Losacco, M. C. , de Almeida, C. F. T. , Hijo, A. H. T. , Bargi‐Souza, P. , Gama, P. , Nunes, M. T. , & Goulart‐Silva, F. (2018). High‐fat diet affects gut nutrients transporters in hypo and hyperthyroid mice by PPAR‐a independent mechanism. Life Sciences, 202, 35–43. 10.1016/j.lfs.2018.03.053 29626530

[fsn32545-bib-0019] Markowiak, P. , & Śliżewska, K. (2017). Effects of probiotics, prebiotics, and synbiotics on human health. Nutrients, 9, 1021. 10.3390/nu9091021 PMC5622781 28914794

[fsn32545-bib-0020] Sahin, N. , Sahin, K. , Onderci, M. , Sarkar, F. H. , Doerge, D. , Prasad, A. , & Kucuk, O. (2006). Effects of dietary genistein on nutrient use and mineral status in heat‐stressed quails. Experimental Animals, 55, 75–82. 10.1538/expanim.55.75 16651689

[fsn32545-bib-0021] Smith, D. E. , Clémençon, B. , & Hediger, M. A. (2013). Proton‐coupled oligopeptide transporter family SLC15: Physiological, pharmacological and pathological implications. Molecular Aspects of Medicine, 34, 323–336. 10.1016/j.mam.2012.11.003 23506874 PMC3602806

[fsn32545-bib-0022] Statovci, D. , Aguilera, M. , MacSharry, J. , & Melgar, S. (2017). The impact of western diet and nutrients on the microbiota and immune response at mucosal interfaces. Frontiers in Immunology, 8, 838. 10.3389/fimmu.2017.00838 28804483 PMC5532387

[fsn32545-bib-0023] Thompson, B. , Amoroso, L. , & C.A.B. International, Food and Agriculture Organization of the United Nations , Eds. (2014). Improving Diets and Nutrition: Food‐Based Approaches. CABI; Food And Agriculture Organization Of The United Nations.

[fsn32545-bib-0024] Tontisirin, K. , Nantel, G. , & Bhattacharjee, L. (2002). Food‐based strategies to meet the challenges of micronutrient malnutrition in the developing world. Proceedings of the Nutrition Society, 61, 243–250. 10.1079/PNS2002155 12133206

[fsn32545-bib-0025] Torelli Hijo, A. H. , Coutinho, C. P. , Alba‐Loureiro, T. C. , Moreira Leite, J. S. , Bargi‐Souza, P. , & Goulart‐Silva, F. (2019). High fat diet modulates the protein content of nutrient transporters in the small intestine of mice: Possible involvement of PKA and PKC activity. Heliyon, 5, e02611. 10.1016/j.heliyon.2019.e02611 31667423 PMC6812199

[fsn32545-bib-0026] Tremmel, M. , Gerdtham, U.‐G. , Nilsson, P. M. , & Saha, S. (2017). Economic burden of obesity: A systematic literature review. International Journal of Environmental Research and Public Health, 14, 435. 10.3390/ijerph14040435 PMC5409636 28422077

[fsn32545-bib-0027] Vásquez‐Velásquez, C. , Gasco, M. , Fano‐Sizgorich, D. , & Gonzales, G. F. (2020). Inflammatory pathway employed by Red Maca to treat induced benign prostatic hyperplasia in rats. Andrologia, 52, e13516. 10.1111/and.13516 31989657

[fsn32545-bib-0028] Vecera, R. , Orolin, J. , Skottová, N. , Kazdová, L. , Oliyarnik, O. , Ulrichová, J. , & Simánek, V. (2007). The influence of maca (*Lepidium meyenii*) on antioxidant status, lipid and glucose metabolism in rat. Plant Foods for Human Nutrition, 62, 59–63. 10.1007/s11130-007-0042-z 17333395

[fsn32545-bib-0029] Wang, S. , & Zhu, F. (2019). Chemical composition and health effects of maca (*Lepidium meyenii*). Food Chemistry, 288, 422–443. 30902313 10.1016/j.foodchem.2019.02.071

[fsn32545-bib-0030] Willett, W. C. , Koplan, J. P. , Nugent, R. , Dusenbury, C. , Puska, P. , & Gaziano, T. A. (2006). Disease Control Priorities in Developing Countries. World Bank.

[fsn32545-bib-0031] Zha, R. , Ge, E. , Guo, L. , Gao, Q. , Lin, Q. , Zhou, W. , Jin, X. , Xie, W. , Yin, H. , & Liu, T. (2021). A newly identified polyunsaturated macamide alleviates dextran sulfate sodium‐induced colitis in mice. Fitoterapia, 152, 104916. 10.1016/j.fitote.2021.104916 33945874

[fsn32545-bib-0032] Zhu, H. , Hu, B. , Hua, H. , Liu, C. , Cheng, Y. , Guo, Y. , Yao, W. , & Qian, H. (2020). Macamides: A review of structures, isolation, therapeutics and prospects. Food Research International, 138, 109819. 33288191 10.1016/j.foodres.2020.109819

[fsn32545-bib-0033] Zwarycz, B. , & Wong, E. A. (2013). Expression of the peptide transporters PepT1, PepT2, and PHT1 in the embryonic and posthatch chick. Poultry Science, 92, 1314–1321. 10.3382/ps.2012-02826 23571341

